# Meeting the governance challenges of integrated health and social care: Josephine Exley

**DOI:** 10.1093/eurpub/ckac129.471

**Published:** 2022-10-25

**Authors:** J Exley, R Glover, M McCarey, S Reed, A Ahmed, H Vrijhoef, T Manacorda, E Stewart, N Mays, E Nolte

**Affiliations:** 1 Health Service Research and Policy, LSHTM, London, UK; 2 The Nuffiled Trust, London, UK; 3 Panaxea, Amsterdam, Netherlands; 4 School of Social Work and Social Policy, University of Strathclyde, Glasgow, UK

## Abstract

**Background:**

Many countries are experimenting with novel ways of organising and delivering more integrated health and social care. Governance is relatively neglected as a focus of attention in this context but addressing governance challenges is key for successful collaboration.

**Methods:**

Cross-country case analysis involving document review and semi-structured interviews with 27 local, regional and national level stakeholders in Italy, the Netherlands and Scotland. We used the Transparency, Accountability, Participation, Integrity and Capability (TAPIC) framework to structure our analytical enquiry to explore factors that influence the governance arrangements in each system.

**Results:**

Governance arrangements ranged from informal agreements in the Netherlands to mandated integration in Scotland. Novel service models were generally participative involving a wide range of stakeholders, including the public, although integration was seen to be driven, largely, from a health perspective. In Italy and Scotland some reversion to ‘command & control’ was reported in response to the imperatives of the Covid-19 pandemic. Policies, budgets, auditing and reporting systems that are clearly aligned at all levels were seen to help with implementing innovations in service organisation. Where alignment was lacking, cooperation and integration was suboptimal, regardless of whether governance arrangements were statutory or not. There was wide recognition of the importance of buy-in. Enablers of greater engagement included visible leadership, time and long-standing working relationships. Lack of suitable indicators and openness to data sharing to measure integration hindered working relationships and thus the successful delivery of integrated services.

**Conclusions:**

Our study provides important insights into how to more effectively and efficiently govern service delivery structures within care systems. We will discuss approaches to governance that help support more resilient integrated care systems.

**Key messages:**

• Different governance arrangements face common challenges to greater integration of care. Enablers include strong leadership, inclusivity and openness to work across traditional boundaries.

• Meeting the governance challenges of integrated health and social care requires clear lines of accountability, aligned policies, budgets and reporting systems.

